# Novel technique for the ultra-sensitive detection of hazardous contaminants using an innovative sensor integrated with a bioreactor

**DOI:** 10.1038/s41598-024-63631-6

**Published:** 2024-06-04

**Authors:** Aleksandra Orzechowska, Anna Czaderna-Lekka, Martin Trtílek, Renata Szymańska, Agnieszka Trela-Makowej, Katarzyna Wątor

**Affiliations:** 1 Faculty of Physics and Applied Computer Science, AGH University of Krakow, al. A. Mickiewicza 30, 30-059 Kraków, Poland; 2 Department of Machine Learning, Faculty of Informatics and Communication, University of Economics in Katowice, 1 Maja 50, 40-287 Katowice, Poland; 3 Photon Systems Instruments, Průmyslová 470, 664 24 Drásov, Czech Republic; 4 Faculty of Geology, Geophysics and Environmental Protection, AGH University of Krakow, al. A. Mickiewicza 30, 30-059 Kraków, Poland

**Keywords:** Antifouling coatings, Bioreactor, Chlorophyll fluorescence spectroscopy, Microalgae, Toxicity, Ultrasensitive sensor, Biophysical methods, Experimental organisms, Microbiology techniques, Sensors and probes, Biological techniques, Ecology, Freshwater ecology, Environmental monitoring, Environmental impact

## Abstract

This study introduces an evaluation methodology tailored for bioreactors, with the aim of assessing the stress experienced by algae due to harmful contaminants released from antifouling (AF) paints. We present an online monitoring system equipped with an ultra-sensitive sensor that conducts non-invasive measurements of algal culture's optical density and physiological stage through chlorophyll fluorescence signals. By coupling the ultra-sensitive sensor with flash-induced chlorophyll fluorescence, we examined the dynamic fluorescence changes in the green microalga *Chlamydomonas reinhardtii* when exposed to biocides. Over a 24-h observation period, increasing concentrations of biocides led to a decrease in photosynthetic activity. Notably, a substantial reduction in the maximum quantum yield of primary photochemistry (F_V_/F_M_) was observed within the first hour of exposure. Subsequently, we detected a partial recovery in F_V_/F_M_; however, this recovery remained 50% lower than that of the controls. Integrating the advanced submersible sensor with fluorescence decay kinetics offered a comprehensive perspective on the dynamic alterations in algal cells under the exposure to biocides released from antifouling coatings. The analysis of fluorescence relaxation kinetics revealed a significant shortening of the fast and middle phases,  along with an increase in the duration of the slow phase, for the coating with the highest levels of biocides. Combining automated culturing and measuring methods, this approach has demonstrated its effectiveness as an ultrasensitive and non-invasive tool for monitoring the physiology of photosynthetic cultures. This is particularly valuable in the context of studying microalgae and their early responses to various environmental conditions, as well as the potential to develop an AF system with minimal harm to the environment.

## Introduction

Bioreactors have firmly established themselves as indispensable tools in modern biotechnology. Serving as platforms, they enable the cultivation of diverse microorganisms under optimized, tightly controlled conditions, thereby meeting the escalating demands of biotechnological applications^1,2^. Photosynthetic microorganisms, particularly algae, have become a focal point of extensive research due to their potential applications, which range from biofuel production^3,4^ to wastewater treatment^5–7^, carbon dioxide sequestration^8–10^, development of nutritional supplements^11,12^, and even the production of novel drugs and therapies in pharmaceuticals^13–15^. To ensure optimal growth, it is essential to strike a balance: fostering high growth rates while efficiently managing energy consumption and related operational costs. Achieving this equilibrium is complex, requiring a profound understanding of the myriad parameters influencing microbial growth. These include aspects such as nutrient availability, light exposure, and potential toxic challenges in the environment^16^. Traditional monitoring methods- such as measuring temperature, pH, redox potential, and light intensity- though crucial^17,18^, may not provide a comprehensive view of the health, productivity, and stress responses of the cultivated organisms.

Recent advancements have highlighted the importance of continuous, real-time monitoring in bioreactors^19–23^. These techniques offer in-depth insights into the well-being and potential stress factors affecting cultured organisms^22^. Among these, chlorophyll fluorescence is one of the most valuable techniques for noninvasively testing the physiological state of photosynthetic samples^24–29^. A standout method in this context is the chlorophyll Q^–^_A_ reoxidation kinetics^30,31^. When paired with ultra-sensitive sensors, this approach can provide detailed insights into the photosynthetic electron transport processes, particularly within photosystem II (PSII) (see^32^). Such innovative combination represents a significant advancement in bioreactor monitoring, a development of particular importance in addressing the environmental challenges posed by antifouling (AF) coatings used in aquatic environments.

In white and industrial biotechnology, biofouling plays a crucial role. Fouling control strategies in algal bioreactors can involve the delivery of AF agents or coatings in various ways. The most common method includes suspending biocides in the bioreactor medium. Furthermore, they can be entrapped in polymeric compounds, delivered as a thin biofilm in membrane bioreactors, or immobilized using various types of carriers (e.g., polymers, porous materials, or organic-derived composites) and introduced into the bioreactors^33–35^. Antifouling coatings, while essential for preventing biofouling on submerged structures, often contain compounds that can be detrimental to aquatic ecosystems^36,37^. The release of toxic substances from these coatings, such as copper, zinc, and organotin compounds into the water bodies is a growing concern. These coatings often incorporate hazardous or toxic substances (biocides) to deter attachment and hinder the growth of fouling organisms, thereby preventing biofouling^36,38^. Common biocides include copper and zinc compounds, organotin compounds, and booster biocides^37,39^. For example, copper, widely used in AF paints, is often present as cuprous oxide or copper thiocyanate, releasing copper ions into the water to inhibit the growth of various organisms^40–45^. Zinc-based compounds, such as zinc pyrithione, zinc oxide, or zineb, release zinc ions that also have inhibitory effects on fouling organisms^46–48^. Booster biocides, secondary additives like irgarol or diuron, enhance the effectiveness of primary biocides like copper^49^. Antifouling paints may also contain an acrylic binder, rosin, inorganic pigments for color, as well as thixotropes and anti-sagging agents for stability and sag resistance^49^. These contaminants can affect not only the fouling organisms they are intended to deter but also a wide range of non-target species, including algae, which play a vital role in aquatic ecosystems^50,51^.

The ability to monitor the effects of hazardous contaminants accurately and non-invasively is therefore of paramount importance. Employing advanced bioreactor technologies, such as the chlorophyll *a* fluorescence measurement technique, allows for non-invasive, automatic, and real-time monitoring of the effects of risky pollutants. This automated approach is invaluable for accurately assessing the impact of AF substances, extending its utility on aquatic organisms. The insights gained are crucial for developing strategies to mitigate the negative environmental impacts of AF coatings, thereby ensuring both the effectiveness of these coatings and the protection of aquatic life.

In this study, we present a novel non-invasive technique for automatically measuring chlorophyll *a* fluorescence using an ultra-sensitive sensor. This method enables the tracking of parameters indicative of algal culture growth and well-being, which can be influenced by AF coatings. By seamlessly integrating the sensitive sensor system with a bioreactor, our goal is to monitor chlorophyll Q^–^_A_ reoxidation kinetics in real-time. This innovative approach represents a significant advancement that can be utilized in aquatic culture management, particularly in addressing hazardous contaminants. Notable enhancements to the measuring head include the incorporation of a flow-through cuvette and a peristaltic pump, facilitating continuous and non-invasive culture transfer to the measuring head. This methodology not only enhances our ability to monitor algal well-being and growth non-invasively but also unlocks new possibilities in biotechnology and ecological safety, all while minimizing disturbance to the organisms under study.

## Materials and methods

### Hazardous and toxic contaminants released from antifouling coatings

The exact composition of the coatings remains undisclosed, as it is protected by the company's confidentiality policies. According to the manufacturer's specifications, the coatings we evaluated in this study are composed of an acrylic binder, rosin, inorganic coloring pigments, thixotropic agents, anti-sagging polyamide pigments, an algaecide (zineb) and copper oxide biocide, with talc included as well. In this study, we used four types of AF coatings assigned as follows: AF1, AF2, AF3, and AF4. The copper oxide content remained consistent across all studied coatings. However, zineb concentrations varied: 12.8% in AF1, 8.6% in AF2, and 4.3% in AF3. Notably, the AF4 coating was zineb-free.

### Determination of elements released from antifouling coatings

To determine elemental content of the AF paints, an inductively coupled plasma optical emission spectrometry (ICP-OES) was used. After a 24-h exposure of the algae to coatings, 10 mL of the algal suspension was homogenized and filtered (0.45 μm, Merck Millipore). The debris remaining on the filter was then dissolved in concentrated HNO_3_ (J.T. Baker, Avantor, Instra-Analyzed) and diluted with ultrapure water to a final volume of 10 mL. The samples were measured with an ICP-OES optical emission spectrometer Perkin–Elmer Optima 7300 DV using analytical lines at 327.393 nm for Cu and 206.200 nm for Zn. The following operating parameters were used: plasma argon 15 L/min, auxiliary argon 1.5 L/min, nebulizer argon 0.8 L/min, RF-power 1300 W and pump rate 1.5 L/min. We used the ICP Multielement Standard Solutions VI (Merck) for the preparation of calibration solutions. To standardise the nebulization conditions of the standard and sample solutions, the standard solutions were replenished with an addition of the mineralizing acids at concentrations equal to those in the analysed samples. All analyses were carried out in three replications, and the average values have been reported along with expanded uncertainty. Quality control of the conducted analyzes included measurement of blank samples and certified reference material (TMDA 64.3, Environment and Climate Change Canada).

### Algae cultivation and the exposure to antifouling coatings

**Figure 1:**
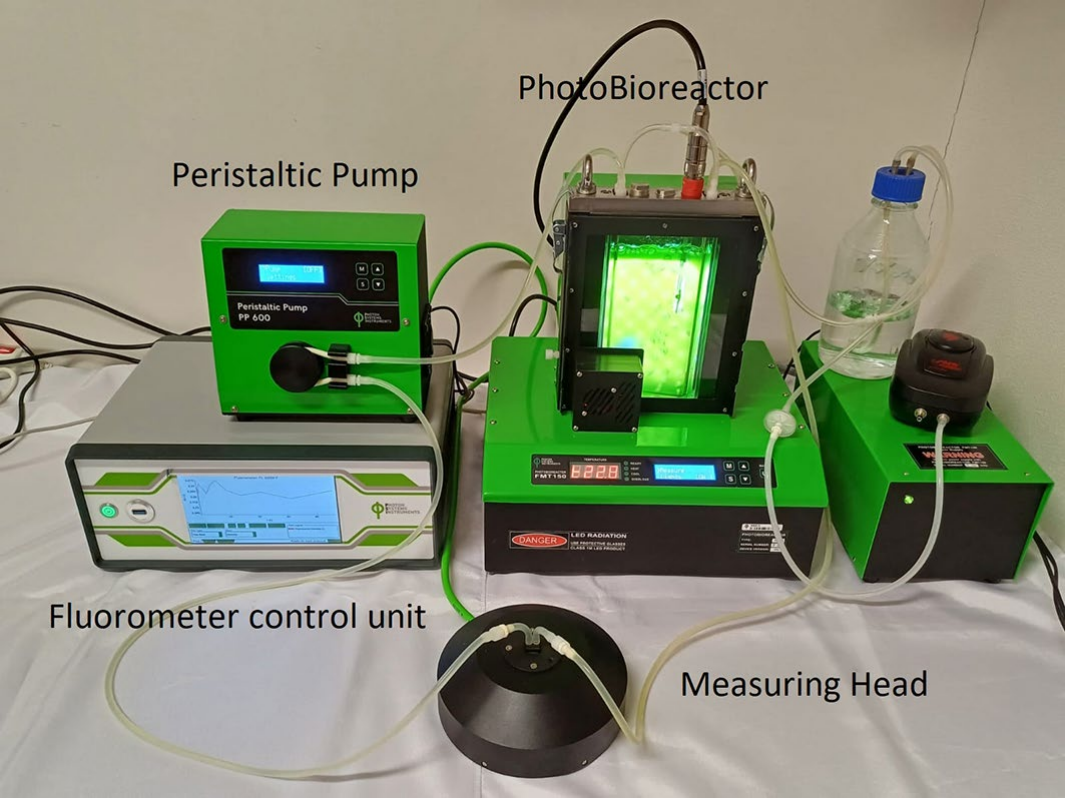
The figure illustrates the advanced algae online monitoring system (Photon Systems Instruments, Drásov, Czech Republic). At its core is the modified measuring head, integrated with a flow-through cuvette, a peristaltic pump, and a fluorometer control unit. This configuration is shown connected to the photobioreactor, demonstrating the continuous and automated transfer of the algae culture to the measuring head.

The wild-type strain of *Chlamydomonas reinhardtii* (*C. reinhardtii*) (CCALA 928) was obtained from the Autotrophic Organisms collection at the Centre of Phycology, Institute of Botany of the AS CR, Trebon, Czech Republic. The algae were cultivated in a flat panel Photobioreactor PBR FMT 150/400-RW PSI (Photon Systems Instruments, Drásov, Czech Republic), a process detailed in^52^ in Bold's basal medium^53^ at a temperature of 25 °C (Fig. 1). A porous sparger was incorporated to maintain a steady airflow. High-intensity LEDs, located on one side of the reactor, ensured homogenous illumination. The control unit of the instrument allowed for adjustments in light intensity, with the LEDs emitting a light intensity of 150 μmol m^−2^ s^−1^. Cultivation vessel capacity was 0.4 L. Temperature inside the system was continuously monitored. The *C. reinhardtii* cultures were exposed to AF coatings (3 × 1.5 × 0.4 cm) from the beginning of the experiment for a duration of 24 h. Throughout this period, the coatings remained continuously submerged, with measurements initiated from the very start and conducted at 15-min intervals. This method was employed to carefully capture the dynamics of subtle changes in the physiological responses of the algae at the earliest possible stage of exposure. The optical density of microalgae was consistently maintained at 0.3.

### Algal-based bioreactor equipped with ultrasensitive sensor

An online algae monitoring system was developed through the adaptation of a dual modulated fluorometer FL6000 for direct integration with a photobioreactor. The system was equipped with submersible module for Q^–^_A_reoxidation, rendering the combination of a fluorometer and a bioreactor entirely unique. This module exhibits exceptional sensitivity, enabling it to detect disturbances within the photosynthetic apparatus at the earliest possible stages. Enhancements to the measuring head included the addition of a flow-through cuvette and a peristaltic pump, facilitating the continuous transfer of the culture to the measuring head (Fig. 1). The cultured suspension was routinely and automatically transferred to the measuring cuvette for analysis, following a pre-established protocol. Post-measurement, the sample was redirected back to the bioreactor, and a fresh sample was introduced into the cuvette. This non-invasive approach ensures the integrity of the samples. Additionally, consistent circulation of the sample during measurements was maintained to uphold the necessary environmental conditions for the cultured organisms. Software enhancements were made to enable automated measurements at predetermined intervals. For precise results, the measuring head was enclosed in a black box throughout the experiments, ensuring the sample remained in complete darkness, which is crucial for dark-condition measurements.

### Measurements of flash-induced chlorophyll fluorescence relaxation kinetics

To examine the reduction and oxidation kinetics of Q_A_, the primary quinone acceptor of photosystem II (PSII), chlorophyll fluorescence and its relaxation in the dark were measured with the dual-modulation LED kinetic fluorometer^54^ (Photon Systems Instruments, Drásov, Czech Republic). Cultures were incubated with coatings at room temperature before initiating fluorescence measurements. Four measuring flashes (4 μs separated with 200 μs intervals, wavelength 620 nm) were applied to determine minimum fluorescence in the dark (F_0_). Samples were excited with a 30 μs red actinic flash from a LED that peaks at 639 nm and prompt fluorescence was measured for 1 min on a logarithmic time scale. Fluorescence emission transients were monitored at 15 min intervals for 24 h. A 2 mL aliquot of *C. reinhardtii* cells was taken at the indicated time points and measurements were performed on the culture directly at an optical density of 0.3. All experiments were carried out in complete darkness. Flash-induced fluorescence decays were analyzed using Origin Professional software version 2019b (Origin-Lab; Northampton, MA, USA).

### Measurements of maximum quantum yield of photosystem II photochemistry

The impact of AF coatings on the photosynthetic activity of *C. reinhardtii* cells was evaluated using in vivo chlorophyll *a* fluorescence. Saturating and measuring light intensities were set at 3000 and 0.05 µmol (photons) m^−2^ s^−1^, respectively. Light was supplied by blue (455 nm) and red (630 nm) light-emitting diodes (LEDs). The maximum quantum yield of PSII photochemistry was expressed as F_V_/F_M_, where F_V_ = F_M_ − F_0_ represents variable fluorescence. F_0_ and F_M_ are the minimum and maximal fluorescence levels, respectively, recorded in the dark-adapted state^55^.

### Statistical analysis

The statistics were analyzed, and the data were evaluated using Origin Professional software version 2019b (Origin-Lab; Northampton, MA, USA). The determination of statistically significant differences in the evaluated time constants between the fluorescence decay kinetics of algal cultures exposed to AF coatings was conducted using the Fisher test.

## Results

### Bioaccumulation of elements in algal cells

The toxicity of hazardous contaminants released from AF coatings was determined using the ICP-OES technique. Four different materials were utilized, containing Cu_2_O and zineb as the primary biocides. The ICP-OES analysis aimed to ascertain the concentration of Cu^2+^ and Zn^2+^, which are the main constituent elements of the biocides, specifically copper dioxide and zineb incorporated into AF paints. The average copper concentration in *C. reinhardtii* cultures exposed to AF coatings ranged from 4.15 ± 0.29 to 4.38 ± 0.25 mg/L. The zinc content in the algal cultures was highest for AF1 coating (1.70 ± 0.20 mg/L) and decreased for AF2 (0.55 ± 0.05 mg/L), AF3 (0.42 ± 0.04 mg/L), and AF4 (0.38 ± 0.04 mg/L) coatings. The bioaccumulation of iron in algal suspensions was also confirmed, with the iron level in *C. reinhardtii* cells being consistent at 0.25 ± 0.03 mg/L.

### Non-invasive chlorophyll fluorescence measurements of *Chlamydomonas reinhardtii* cells under exposure to antifouling coatings in algal-based bioreactor

**Figure 2:**
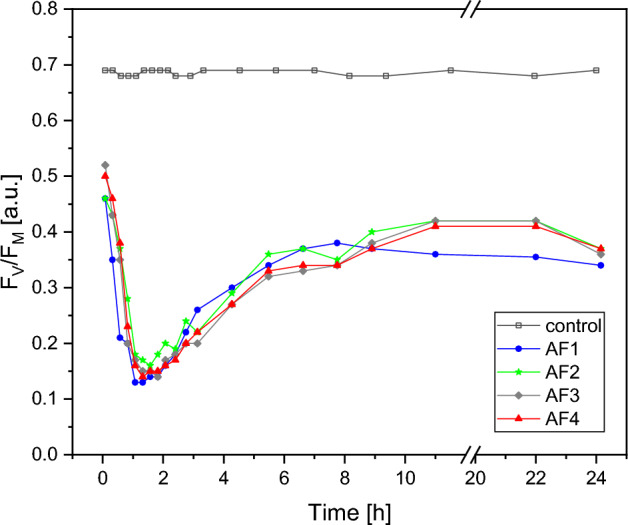
Changes in F_V_/F_M_ measured at 15-min intervals over the initial 24 h of *C. reinhardtii* exposure to antifouling paint coatings (AF1, AF2, AF3, AF4) with control group not exposed to any coating.

To elucidate the impact of AF coatings on the activity of PSII, we evaluated the maximum quantum yield of PSII photochemistry, denoted as F_V_/F_M_ (Fig. 2). For all tested AF coatings, a significant inhibitory effect on the photosynthetic activity of *C. reinhardtii* cells was observed. The most pronounced deviations were evident within the initial hour post exposure. Subsequent to this initial decline, a notable increase in F_V_/F_M_ values was observed, reaching its maximum at the 11-h mark. However, this recovery was still 50% lower than that of the controls. Beyond this point, a gradual decline in F_V_/F_M_ ensued.

Amongst the AF coatings studied, those enriched with elevated concentrations of copper and zinc ions (designated as AF1) exhibited the most severe impact. For AF1, the rate of decline in F_V_/F_M_ was the most rapid, and from the 11-h mark onward, AF1 persistently recorded the lowest F_V_/F_M_ values in comparison to other (AF2, AF3, AF4) coatings. Analysis of these coatings showed that the period between 11 and 22 h post exposure marked a moderated reduction in F_V_/F_M_ values relative to AF1. By the end of 24 h, this difference had narrowed, with F_V_/F_M_ values being just 8% below the controls.

**Figure 3:**
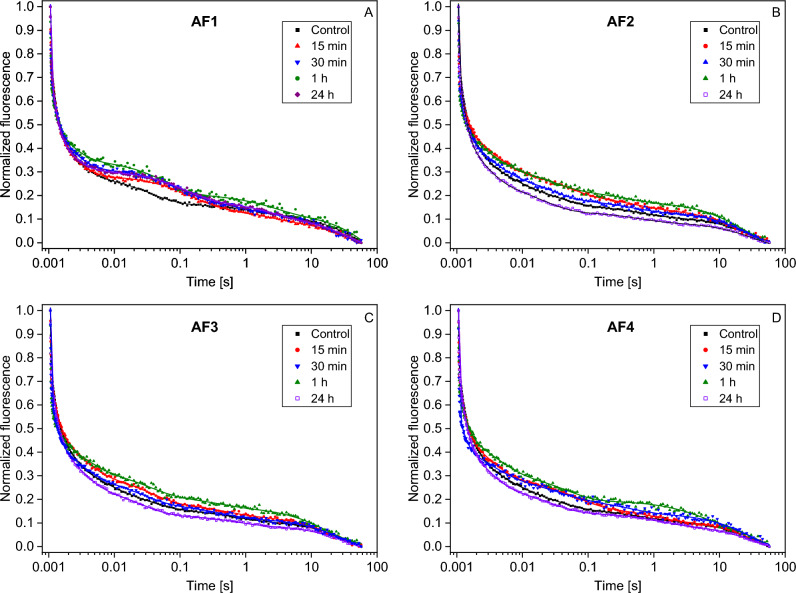
Normalized fluorescence kinetics which were measured for *C. reinhardtii* cells exposed to AF coatings: AF1 (**a**), AF2 (**b**), AF3 (**c**), and AF4 (**d**). The traces represent different algal exposure times, each with their corresponding fitted curves (Eq. 1). To enhance clarity, only specific kinetics from among more than 20 traces are displayed.

Q^–^_A_ reoxidation kinetics offer a more detailed understanding of the electron transport processes and the dynamic behavior of PSII under varying environmental conditions. Therefore, we investigated the alterations in chlorophyll Q^–^_A_ reoxidation kinetics in the presence of biocides released from AF coatings. Our results emphasize the significant impact of these biocides on the photosynthetic processes occurring within PSII. Measurements were conducted over a span of 24 h, taken at 15-min intervals. Initial observations indicated that the AF coatings had a notable impact on the reoxidation kinetics of Q^–^_A_. Detailed kinetics for each AF treatment were represented with distinct patterns, as seen in Fig. 3. Each coating, namely AF1 (Fig. 3a), AF2 (Fig. 3b), AF3 (Fig. 3c), and AF4 (Fig. 3d), demonstrated unique effects on the physiological processes of the *C. reinhardtii* cultures. To elucidate the dynamics of chlorophyll relaxation changes in algal cultures subjected to AF coatings, we evaluated the fluorescence decay kinetics using the multi-exponential function (Eq. 1):

1 $$F\left( t \right) = y_{0} + \mathop \sum \limits_{i} \left[ {A_{i} \cdot\exp \left( { - t/t_{i} } \right)} \right].$$

**Figure 4:**
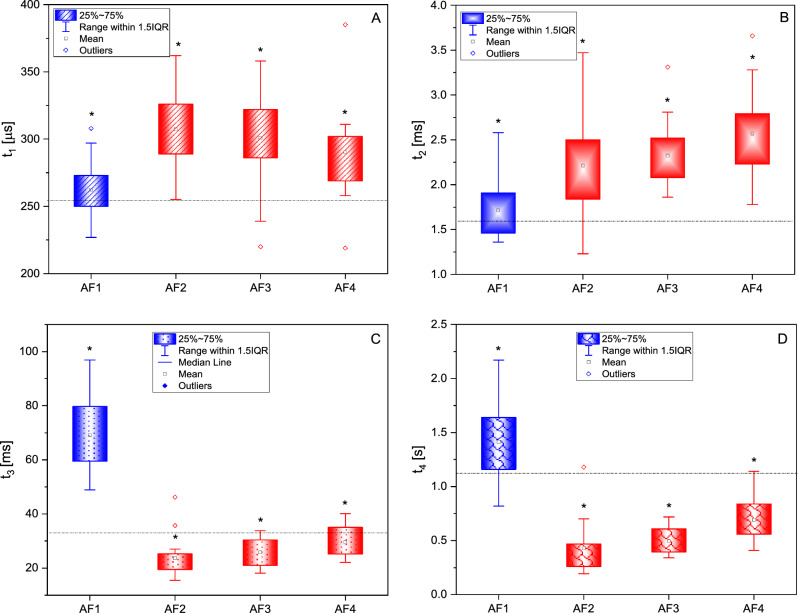
Time constants determined from fitting fluorescence decay curves using Eq. 1. Fluorescence measurements were taken from *C. reinhardtii* culture exposed to AF coatings (AF1, AF2, AF3, AF4) at 15-min intervals over the first 24 h. Each estimated time constant—t_1_ (**a**), t_2_ (**b**), t_3_ (**c**), and t_4_ (**d**) is presented as the box-whisker plots (interquartile spans), showing mean and outliers at 95% confidence level and illustrate their statistical variability (*). The dashed line indicates the control sample. Statistical analysis was performed using Fisher test. Each time constant represents at least 20 measurements for each of the evaluated AF coatings.

In this function, *F(t)* is a fluorescence value at time *t, t*_*i*_ represents the characteristic time constant, *y*_*0*_ corresponds to the amplitude at the end of the *F(t)* decay, and *A*_*i*_ denotes the amplitude. Interestingly, our analysis identified the phases (Fig.4) with time constants of approximately 0.25–0.31 ms (Fig. 4a), 1.70–2.55 ms (Fig. 4b), 23–70 ms (Fig. 4c), and 0.43–1.40 s (Fig. 4d). A component with a time constant of about 18.15±3.65 s was also determined. Three of these phases align with the established fast (Fig. 4a), middle (Fig. 4b), and slow (Fig. 4d) phases. Also, the additional phase (Fig. 4c) seems unique to *C. reinhardtii*, as it is not observed in plants and cyanobacteria.

When analyzing the fluorescence patterns for *C. reinhardtii* exposed to AF coatings, we found that subsequent phases displayed significant changes depending on the amount of biocides incorporated into the AF coatings. Notably, under exposure of AF coating that contained the highest concentration of biocidal substances (AF1), our data showed that the time constants considerably diverged from those evaluated for the AF2, AF3, and AF4 coatings. This divergence was evident in the reduction of the time constants for the shortest phases (Fig. 4a,b) and the rise of the time constants for the other two phases as shown in (Fig. 4c,d). In the analysis of AF coatings with reduced (AF2, AF3) or no zineb (AF4) content, a shortening (Fig. 4a) and a lengthening (Fig. 4b–d) of phases were observed corresponding to the decrease in zineb concentration.

## Discussion

The cultivation of microalgae in photobioreactors and the advancements in photosynthetic engineering have become increasingly prominent. Achieving optimal growth conditions is crucial for maximizing the capabilities of microalgae-based systems. By integrating photosynthetic engineering with advanced monitoring techniques, significant environmental challenges can be addressed^56–58^. In this study, we introduce a new system that operates automatically and utilizes ultrasensitive detectors to measure the fluorescence of photosynthetic samples exposed to AF coatings. Although the fluorescence technique is widely recognized as one of the most suitable and accurate methods for assessing the photosynthetic activity of the sample^26,59–61^, and the effect of antifouling paints on chlorophyll fluorescence kinetics is well known^38,62,63^, our work is pushing the boundaries of sensitivity, enabling continuous evaluation of the influence of AF paints on aquatic organisms. We proposed a novel system that can automatically measure chlorophyll *a* fluorescence and, through the implementation of a submersible Q^–^_A_ reoxidation module, also monitor the dynamic changes occurring in the photosynthetic apparatus of microalgae. This research showed, the distinctive advantage of the proposed system lies in its provision for continuous, real-time measurements, significantly enhancing its utility in dynamic studies of organisms subjected to hazardous contaminants released from AF coatings. The instrument's design enables the modification of parameters such as temperature, light intensity, and optical density. This provides unparalleled control and versatility, also allowing for the accurate simulation of environmental conditions. These capabilities enable the accurate replication and precise simulation of environmental conditions, allowing for the detailed observation of their effects on biological specimens at an exceptional early stage. While various studies have showcased the capabilities of the FL6000 fluorometer^64–66^ and the FMT-150 bioreactor^67,68^ in distinct contexts, these tools are not inherently suited for analyzing the dynamic biological activities in cultures as required for our research objectives. This is because the FMT-150 bioreactor lacks the Q_A_'s ultra-sensitive reoxidation module, a crucial component for such analyses. In our setup this module is engineered for highly sensitive detection without necessitating manual sample introduction. This feature is pivotal for identifying physiological changes in cells at the earliest stages of stress exposure. Additionally, conventional fluorescence parameters like F_0_, F_V_, F_M_, and NPQ lack the sensitivity necessary for our research, especially for assessing the toxicity effects of antifouling paint on the very early stage. The Q^–^_A_reoxidation module is crucial for delivering the required sensitivity needed to detect the effects of toxicity from antifouling paints. Currently, there is no existing submersible module for Q^–^_A_reoxidation, rendering the combination of double modulated fluorometer and a bioreactor entirely unique. Such a module is extremely sensitive to detect any disturbances in the photosynthetic apparatus. This sensitivity is particularly crucial for detecting the antifouling effect at the earliest stage of fouling. The development of new, more environmentally friendly antifouling paints that effectively protect surfaces susceptible to fouling (such as ship or boat hulls) while having minimal environmental impact requires more sensitive instrumentation and methodologies. Our work presents such state-of-the-art instrumentation and methodology.

In this research, we assessed the photosynthetic activity of *C. reinhardtii* cultures exposed to copper-based AF coatings formulated with or without zineb. The efficiency of these coatings was assessed by measuring the release of the main biocidal agents, copper and zinc ions, from the coating surfaces into the culture medium in a bioreactor. The study revealed that after 24 h of exposure, AF coatings significantly inhibited the photosynthetic activity of algal cells, reducing the F_V_/F_M_ to 50% of its initial value. This finding aligns with previous research indicating that copper^69–71^ and zinc^72^ are among the most toxic biocides affecting freshwater algae. The toxic effects on *C. reinhardtii* cells became evident at a copper concentration of 6.7 μg/L. Interestingly, in our study, the concentration of copper released from AF coatings was significantly higher, ranging from 4.15 ± 0.29 to 4.38 ± 0.25 mg/L. Copper exposure has a multifaceted impact on *C. reinhardtii*, influencing biochemical, physiological, and growth levels^73^. Interestingly, copper can have contrasting effects, either promoting or inhibiting algal growth, while also affecting trace element uptake^74^. Notably, the addition of Cu can lead to an increased accumulation of Zn and Fe in *C. reinhardtii*. Recent studies on *C. reinhardtii* exposed to environmentally relevant concentrations of copper and zinc have demonstrated that Zn promotes the entry of Cu into the algal cells. This phenomenon may be attributed to the synergistic and antagonistic interactions among trace elements during adsorption and uptake processes, as well as changes in metal speciation in the culture medium^74^. Such interactions have the potential to enhance the toxic effects of copper. Moreover, a recent study by^75^ has demonstrated that as the combined toxicity of copper and zinc increases, extracellular polymeric substances and cell wall functional groups immobilize significant amounts of Cu and Zn. Additionally, *C. reinhardtii* adapts its internal resistance strategies, including starch consumption and the synthesis of chlorophyll and lipids. When exposed to high levels of coexisting Cu and Zn, positive synergistic effects are observed in the reduction of lipid peroxidation and an enhancement in catalase (CAT) activity. This could explain the increased photosynthetic activity observed after 1 h of exposure to AF coatings in our study.

The experiments measuring the maximum quantum yield of photosystem  II (F_V_/F_M_) and Q^–^_A_ reoxidation are both commonly employed in the field of photosynthesis research to evaluate the efficiency of the photosynthetic apparatus, specifically within PSII. However, they address distinct aspects of PSII function. Unlike F_V_/F_M_, which offers a snapshot of the potential efficiency, Q^–^_A_ reoxidation kinetics can quantify actual electron transport rates through PSII. This allows for a more direct and sensitive measurement of photosynthetic activity. Changes in Q^–^_A_ reoxidation kinetics can reveal early stress responses in photosynthetic organisms before any significant changes in F_V_/F_M_ ratios occur. It also detects subtle changes in photosynthetic mechanisms that might not significantly affect F_V_/F_M_ ratios. The analysis of fluorescence kinetics revealed that *C. reinhardtii* cultures were promptly affected by AF coatings. Observable changes in fluorescence traces occurred within the first 15 min of exposure to AF coatings and continued to change every 15 min over 24 h of exposure. The decay of fluorescence consisted of several kinetic phases corresponding to different pathways of Q^–^_A_ reoxidation, which report on forward or backward electron transfer, as discussed in^30,76,77^. To provide reliable interpretations of the chlorophyll fluorescence transients, it is necessary to appropriately evaluate each of the kinetic components. Our analysis revealed the phases: (i) a fast phase related to Q^–^_A_ reoxidation by a secondary quinone acceptor Q_B_, (ii) a middle phase associated with reoxidation by plastoquinone (PQ) binding to the Q_B_ site after the flash, and (iii) a slow phase attributed to Q^–^_A_ reoxidation via charge recombination with the S2 state of the oxygen evolving complex in PSII reaction centers that cannot transfer the electron to the PQ pool^30,78,79^. Interestingly, an additional kinetic phase (several tens of millisecond) was observed, which is characteristic of *C. reinhardtii* but absent or reduced in higher plants and cyanobacteria; the nature of this phase remains unclear^22^. The time constant of the fast phase increased compared to control cells, indicating a significantly slower electron transfer from Q_A_ to Q_B_ under antifouling coatings exposure. Similarly, the time constant of the middle component in algal cells showed a significant increase compared to the control. These changes in electron transfer from Q_A_ to (Q_B_)/Q^-^_B_ and Q_B_-binding are associated with the accumulation of reduced plastoquinone (PQ) in the photosynthetic membrane and a decrease in the apparent equilibrium constant between Q_A_ and Q_B_^80^. In turn, the alterations in the slow phase may suggest disturbances in charge recombination between Q_B_ and the S2 state, likely due to dysfunction on the PSII donor side^29,80^. Since each of these components corresponds to specific electron transport processes within PSII, their yields and the time constants can be used for a comprehensive characterization of primary photosynthetic reactions within PSII under the influence of biocides. In this study, what appears to be particularly interesting is AF1 coating with the highest amount of zineb when compared to the other coatings (AF2, AF3, AF4). This coating exhibited a significant shortening of the fast and middle phases of the fluorescence kinetic traces. Furthermore, for this coating, we also observed a delay of the slow phase by at least 1.7-fold and, specific to *C. reinhardtii*, a delay of the fluorescence decays by at least 2.3-fold. This allows us to identify AF1 coating as the most toxic coating in our study. As previously reported in^81^, the determination of fluorescence time constants is a valuable method used to study the dynamics of the effectiveness of AF coatings on photosynthetic aquacultures.

The technique offers a universal approach that can be applied to various algal or cyanobacterial cultures, bioreactor types, and stress factors. Its key advantage is high sensitivity, making it an effective tool for detecting changes in photosynthetic processes influenced by diverse stress conditions, especially the toxicity of numerous hazardous chemicals. Our proposed system offers also a viable solution for wastewater treatment. Effluents often contain toxic chemicals that can interfere with cell cultures. Therefore, the measuring setup enables the evaluation of the stress response of algal cells under such conditions during the culture development process. Given that the culture can rapidly respond to hazardous agents in incoming wastewater, even at barely detectable concentrations, this capability could be a significant advantage in environmental monitoring and remediation efforts. It is important to note that the Q^–^_A_reoxidation module is not specific and cannot identify the particular type of stress present. However, it does provide insights into the subtle changes in the physiology of the culture during the initial stage of contact with hazardous materials or other unfavorable physicochemical factors (such as temperature, lighting, pH, etc.). These insights are crucial for the development and refinement of antifouling strategies that prioritize both effectiveness and environmental friendliness.

Despite the undeniable advantages of this approach, it is important to consider that laboratory-scale experiments may not always accurately reflect performance at contaminated sites. For instance, in real-world conditions, algae often become acclimated to certain stress factors, a detail frequently overlooked in laboratory studies. Employing an acclimated algae culture in experiments could more effectively bridge the gap between laboratory-scale results and actual treatment conditions. To evaluate the scalability of results from laboratory studies to larger scales, additional experiments are necessary. These experiments should account for factors such as adequate nutrient supply, mass and heat exchange, as well as fluid and gas flow, pressure above the liquid, and the partial pressures of oxygen and CO_2_. Furthermore, scaling up the process requires the use of advanced mathematical modeling and bioinformatic tools to identify bottlenecks and optimize the entire process.

## Conclusions

The integration of advanced culturing and monitoring techniques, particularly automated and non-invasive measurements of the dynamics of chlorophyll *a* fluorescence, has enabled a comprehensive assessment of the impact of hazardous contaminants released from AF paint coatings on the photosynthetic activity of microalgae. This study offers valuable insights into the effects of AF coatings on microalgae photosynthesis, emphasizing the importance of grasping and refining coating formulations for sustainable aquaculture practices aimed at minimizing harmful ecological impacts.

## Data Availability

The datasets used and/or analysed during the current study available from the corresponding author on reasonable request.

## Abbreviations

AF: Antifouling
*C. reinhardtii*: *Chlamydomonas reinhardtii*
F_0_ (F_M_): The minimum (maximum) chlorophyll *a* fluorescence in the dark-adapted state
F_V_/F_M_: The maximum efficiency of photosystem II photochemistry in the dark-adapted state
ICP: Inductively coupled plasma emission spectrometry
PSII: Photosystem II
PQ: Plastoquinone
Q_A_ (Q_B_): Primary (secondary) quinone acceptor of photosystem II
